# Dental microplastics as emerging neurotoxicants: a systematic review on human data

**DOI:** 10.7717/peerj.20829

**Published:** 2026-02-26

**Authors:** Aiah Alkhamees, Dana Salha, Dana Alfailat, Danah Malallah, Dhewy Alazemi, Dunia Al Taharweh, Ebaa Al Borom, Asmaa Uthman, Musab Hamed Saeed, Natheer H. Al-Rawi

**Affiliations:** 1 College of Dental Medicine, University of Sharjah, Sharjah, United Arab Emirates; 2 College of Dentistry, Gulf Medical College, Ajman, United Arab Emirates; 3 College of Dentistry, Ajman University, Ajman, United Arab Emirates; 4 Dept. Oral & Craniofacial Health Sciences, College of Dental Medicine, University of Sharjah, Sharjah, United Arab Emirates

**Keywords:** Microplastics, Bisphenol A, Neurodegenerative diseases, Alzheimer’s disease, Parkinson

## Abstract

**Background:**

Microplastics and compounds linked to plastic have recently emerged as potential contaminants that might affect brain function; nevertheless, results from these investigations have been inconsistent across epidemiological, clinical, and mechanistic research. Whether in people or *in vitro* models, this systematic review sought to compile the most recent data on the link between exposures to microplastics and neurological effects.

**Methods:**

A comprehensive search of PubMed, Scopus, MEDLINE, EBSCO, and ScienceDirect was performed to identify studies published from January 2015 to December 2025. Eligible studies assessed the relationships between microplastics, nanoplastics, or associated chemical markers (*e.g.*, bisphenols, phthalates) and neurological outcomes, including cognitive function, neurodegenerative biomarkers, or neuronal injury mechanisms. Two evaluators independently conducted study screening, data extraction, and quality assessment utilizing the Newcastle–Ottawa Scale for human studies and the ToxRTool for *in-vitro* studies.

**Results:**

Out of 477 records, 18 research fulfilled the inclusion criteria: nine human observational studies, one postmortem analytical study, and eight *in-vitro* mechanistic investigations. Human investigations indicated correlations between elevated internal exposure to microplastics or plastic-associated compounds and altered cognitive function or neurodegenerative biomarkers; yet, all were cross-sectional and failed to demonstrate causality. The postmortem study revealed microplastics buildup in brain tissue, but *in vitro* investigations elucidated molecular mechanisms including oxidative stress, mitochondrial malfunction, autophagy disruption, and protein aggregation that may contribute to neurotoxic consequences. Because of heterogeneity, results were synthesized within exposure and outcome specific subgroups instead of being merged.

**Conclusion:**

Current evidence indicates possible neurological effects of microplastics-related exposures, corroborated by similar molecular pathways in *in-vitro* research and connections identified in human cross-sectional data. Nevertheless, the primarily observational and experimental characteristics of existing studies hinder definitive conclusions about clinical causation. Additional longitudinal, standardized human investigations are required to elucidate dose–response relationships and the applicability of *in-vitro* findings to real-world exposure.

## Introduction

Plastics have become an intrinsic component of human life, with the growing use of plastics in industrial, medical, personal care, and commercial industries. The weathering of plastics by mechanical, biological, or photolytic mechanisms breaks plastics into smaller particles, called microplastics (Hariharan et al., 2022; Bhat, Gedik & Gaga, 2024). Microplastics are defined as small synthetic plastic particles with a size less than 5 millimeters (Marzouk et al., 2019). On average, an individual breathes, drinks, and eats 220,000 particles of microplastics every year (Cox et al., 2019). These particles may pose a threat on the health of both humans and animals, alter ecosystems and food chains, and harm marine life.

Specific to dentistry, two types or sources of exposure to microplastics can occur. Plastic particles may be manufactured to a microscopic size, which is a primary source of exposure. The primary source of microplastics in dentistry is toothpaste that contains microbeads and exfoliating agents (Bashir et al., 2021; Chandran, Vishnu & Warrier, 2021). The secondary source of exposure to microplastics can occur from the disintegration of larger plastic particles to smaller microplastics in the oral cavity. These secondary sources of microplastics in dentistry include the breakdown of resin-based composite restorative materials which degrade in the oral cavity and the microplastics that may be released during finishing and polishing procedures (Chandran, Vishnu & Warrier, 2021; Madhumitha et al., 2022; Mulligan et al., 2021). Primary and secondary sources of microplastics may also occur from other oral health care products, such as toothbrushes, tooth powder, mouthwash, dental floss, and mouth freshener sprays as well (Protyusha, Kavitha & Robin, 2024).

Although the exposure of individuals to microplastics tends to be overlooked, they can lead to the release of toxic chemicals, disrupting the body and resulting in a wide array of adverse effects. Microplastics obtained from dental origins can translocate to distal tissues in the body through the circulatory system (Protyusha, Kavitha & Robin, 2024). Due to the ability of microplastic particles to translocate from their original site of exposure, there is the potential for neurotoxicity (Stapleton, 2021). Very tiny plastic particles, 0.1–10 um in size can reach the brain by crossing the blood-brain barrier (Sincihu et al., 2023). The blood-brain barrier is a network of blood vessels and tissues that protects the brain from toxins and pathogens, and microplastics that bypass it may cause neurologic disorders and diseases (Prüst, Meijer & Westerink, 2020).

Neurodegenerative disorders are projected to surpass cancer and become the second most prevalent cause of mortality in the coming years, as stated by the World Health Organization (Bi et al., 2022). Neurodegenerative disorders are defined by the gradual deterioration of the structure or functionality of neurons. Parkinson’s disease, Alzheimer’s disease, and amyotrophic lateral sclerosis (ALS) are among the most prevalent neurodegenerative disorders, affecting around 15% of the population annually (Zaib et al., 2023; Van Schependom & D’haeseleer, 2023). Doctors and researchers are trying to find preventive measures to protect the aging population from such diseases. The majority of research undertaken on this subject has been focused on animals. However, due to the reported disparities between human and animal oligodendrocytes, animals may not be sufficient for accurately mimicking these disorders (Chesnut et al., 2021). This systematic review will thoroughly investigate the cause–effect relationship between microplastics in dental materials and neurodegenerative diseases in humans, which is currently the subject of contradictory studies. The main goal of this study is to conduct a comprehensive assessment of the available information that evaluates the impact of microplastic exposure from dental materials and therapies on the occurrence of neurodegenerative disorders in individuals. In addition, it is important to ascertain the properties of microplastics used in dental procedures that might potentially impact cognitive function. 

## Materials and Methods

### Protocols

The data collection and analysis for this systematic review were conducted in accordance with the principles outlined in the Preferred Reporting Items for Systematic Reviews and Meta-analyses (PRISMA 2000) statement (Page et al., 2021). The review’s focused question was answered by employing the participant, intervention, comparison, and outcomes (PICO) criteria (Hosseini et al., 2024). The study protocol was filed on the PROSPERO platform, an international prospective register of systematic reviews, with the registration number CRD42023476370 on17/11/2023 (https://www.crd.york.ac.uk/PROSPERO/view/CRD42023476370).

### Search strategy

Five main electronic databases were (PubMed, EBSCO, Scopus, MEDLINE, ScienceDirect) and manual searches were used to find papers that met the eligibility criteria. The search was conducted for the last ten years. The following Medical Subject Headings (MeSH) and keywords were used for the selected database: [Microplastic [mesh terms] OR (microplastics) OR (microbead) OR (nanoplastic) OR (plastic microparticles) OR (microparticle, plastic)) AND (dental materials [mesh terms] OR (dental material) OR (material, dental) OR (dental treatment) OR (composite) OR (resin-based composite) OR (bisphenol A) OR (bis-GMA) OR (bisphenol A-glycidyl methacrylate) OR (BPA) OR (phthalate) OR (toothpaste)) AND (Neurodegenerative disease [mesh terms] OR (Parkinson’s) OR (dementia) OR (Alzheimer’s disease) OR (Alzheimer) OR (neurotoxicity) OR (neurotoxic) OR (cognitive degeneration) OR (cognitive deterioration)] for PubMed database. Search strategies for the remaining databases are included in Table S1.

### Inclusion criteria

Publications were taken into consideration if the following inclusion criteria used to screen and assess the retrieved records was met: (a) clinical trials, case-control, cross-sectional, *in vitro* on human cells, or cohort studies (b) full-text articles, (c) articles published in English, (d) articles published after the year 2010, (e) articles with neurodegenerative diseases as the outcome of interest, (f) articles with reported exposure to dental microplastics.

### Exclusion criteria

Reports were excluded if they were animal studies, review articles, conducted on embryonic cells or embryos, if the population in the study had congenital or hereditary neurological disorders, or if the study was about microplastics that are not related to dental sources. Studies published in languages other than the English language and those published before 2010 were also excluded.

### Focused PICO question

Is there a relation between the exposure of patients to microplastics in dental materials and the development of neurodegenerative diseases?

Participants: general population

Intervention: exposure to dental microplastics

Comparison: cognitively healthy individuals

Outcomes: the development of neurodegenerative diseases

### Selection of studies

All records retrieved from the database searches were imported into Microsoft Excel for organization and duplicate removal. The selection process followed the PRISMA 2020 framework. After removing 67 duplicates, two reviewers independently screened titles and abstracts of 410 records according to the predefined eligibility criteria. Articles unrelated to microplastics (*n* = 324) and studies conducted exclusively on animal models (*n* = 29) were excluded at this stage, leaving 57 records for full-text review.

**Figure 1:**
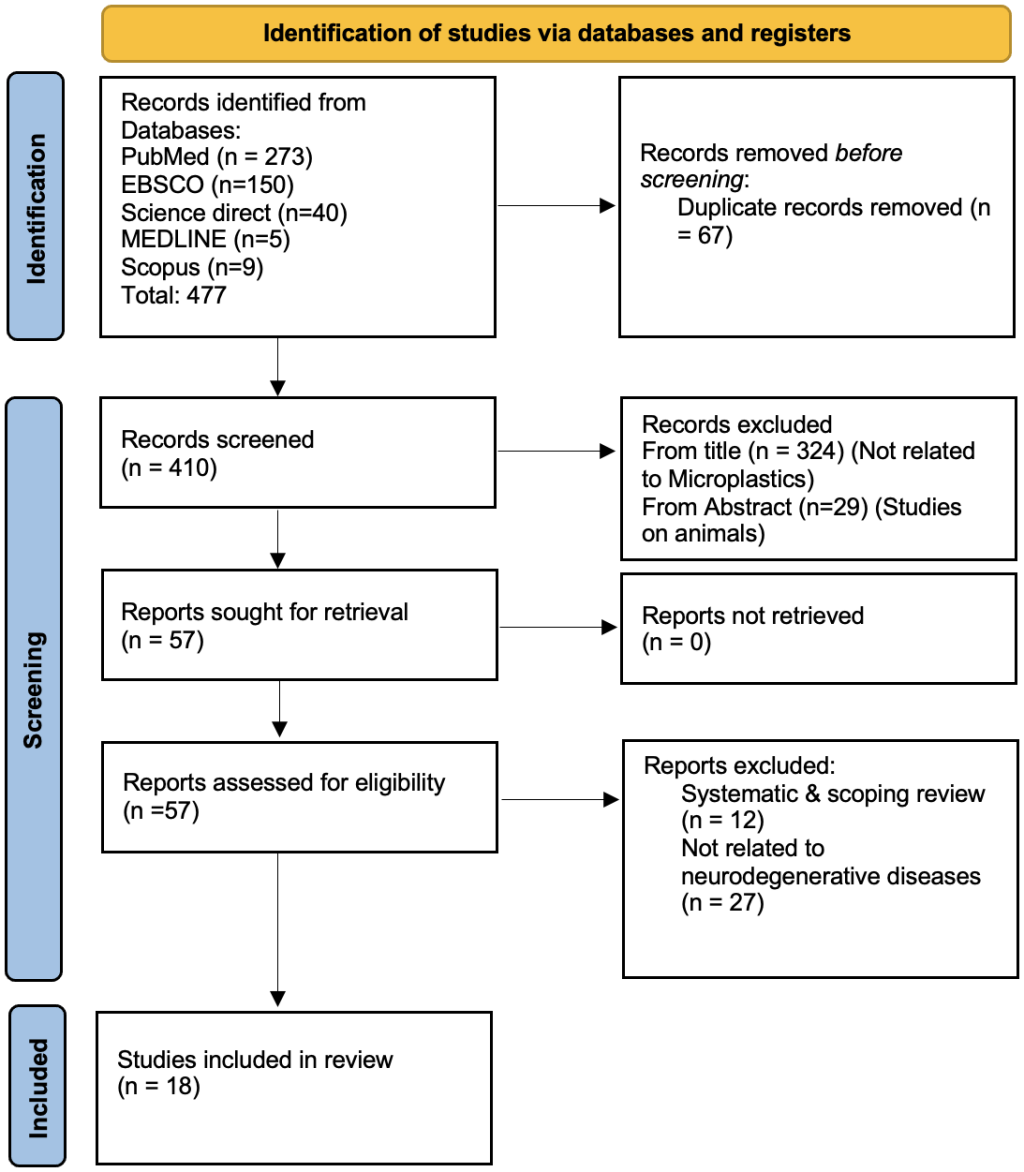
PRISMA model.

Full-text articles were then obtained and independently assessed by the same two reviewers. Reasons for exclusion included being review articles (*n* = 12) or not reporting neurodegenerative outcomes (*n* = 27). Ultimately, 18 studies met the eligibility criteria and were included in the qualitative synthesis (Fig. 1). Inter-rater reliability for title and abstract screening was evaluated using Cohen’s κ, yielding κ = 0.70, indicating substantial agreement. All disagreements at any stage were resolved through group discussion. Data extraction was performed independently by two reviewers using a structured data extraction form developed in Microsoft Excel. The form captured study characteristics, participant demographics, exposure type, outcomes, and key findings. Extracted data were cross-checked for accuracy by a third reviewer, and any inconsistencies were resolved through discussion and consensus among all authors. Final data tables were validated by the senior reviewer prior to analysis. A meta-analysis was not undertaken due to major heterogeneity in study designs, exposure assessments, and outcome measures, along with insufficient standardized quantitative data for effect-size pooling.

### Quality assessment

Quality appraisal was performed separately for clinical and *in-vitro* studies to ensure use of the most appropriate tools for each study type. Human observational studies (cross-sectional, case-control, and prospective designs) were evaluated using the Newcastle–Ottawa Scale (NOS), which assesses methodological quality across three domains: selection, comparability, and outcome (Wells et al., 2000).

In contrast, *in-vitro* cell culture studies were appraised using the Toxicological Data Reliability Assessment Tool (ToxRTool), a validated instrument specifically designed for laboratory toxicology research. The ToxRTool evaluates five domains: test substance identification, test system and cell line characterization, study design, reporting of results, and overall study plausibility (Schneider et al., 2009). All assessments were conducted independently by two reviewers, and final scores were confirmed through consensus Table S2.

### Risk of bias

In most studies we found moderate to high quality. In National Health and Nutrition Examination Survey (NHANES) studies we saw low risk of bias which came from representative sampling, validated exposure assays, and also from strong confounder adjustment (Weng et al., 2022; Shi et al., 2023). Case-control studies did report robust exposure measures although in terms of comparison that was only moderate. Also, we saw in small clinical studies which had a very small sample size with high selection bias present (Magalhães et al., 2019), but in the large pediatric study which used advanced modeling we saw a low overall risk of that (Zheng et al., 2025). Also in all nine *in vitro* cell culture studies we noted that they were very reliable without any restrictions *via* use of ToxRTool which scored between 15 to 18 out of 18, the main issues we did see were that nanoplastic purity and cell passage number weren’t fully reported (Tables 1A & 1B).

**Table 1:** Quality assessment of the included studies.

A. Quality assessment of the included studies using the Newcastle-Ottawa scale.						
**Study**	**Study design**	**Selection**	**Comparability**	**Outcome/ Exposure**	**Total**	**Justification for domain scores**
Weng et al. (2022)	Cross sectional	4	2	3	9	**Selection:** National NHANES sampling frame; validated urinary phthalate biomarkers; objective cognitive tests. ** Comparability:** Adjusted for multiple demographic, lifestyle, and clinical covariates. ** Outcome:** Standardized neurocognitive battery and mixture models with high methodological rigor.
Shi et al. (2023)	Cross sectional	4	2	3	9	**Selection:** NHANES dataset with representative sampling; high-quality BPA/paraben measurement. ** Comparability:** Sex-stratified models; adjustment for major confounders. ** Outcome**: Validated memory tests and advanced mixture modeling.
Zheng et al. (2025)	Cross sectional	4	2	3	9	**Selection:** Very large school-based cohort; well-defined recruitment; standardized exposure measurement. ** Comparability:** Controlled for socioeconomic, demographic, and behavioral confounders. ** Outcome:** Validated cognitive outcomes; robust statistical mixture approaches.
Xie et al. (2024)	Cross sectional	2.5	1	3	6.5	**Selection:** Clinical sample with limited representativeness; CSF microplastic quantification strong. ** Comparability:** Minimal confounder adjustment. ** Outcome:** Valid CSF biomarkers; objective albumin index measures.
Magalhães et al. (2019)	Cross sectional	2.5	1	2.5	6	**Selection:** Small convenience sample; appropriate case definition; MPr measurement valid. ** Comparability:** Limited adjustment for confounders. ** Outcome:** Standard AD/MCI cognitive scales; small sample reduces precision.
Nihart et al. (2025)	Cross sectional postmortum	3.5	1	3	7.5	**Selection:** Autopsy-based sample; detailed tissue MP identification (Py-GC/MS, TEM). ** Comparability:** Minimal control for premortem variables. ** Outcome:** Strong polymer verification; systematic neuropathological assessment.
Landolfi et al. (2017)	Case control	4	1	3	8	**Selection:** Clear case definition (PD diagnosis); spouse controls; validated BPA measurement. ** Comparability:** Limited covariate adjustment. ** Outcome:** Reliable BPA fractionation and lab QA/QC.
He et al. (2025)	Case control	4	1	3	8	**Selection:** Biomarker-defined groups (amyloid+/-); appropriate sampling. ** Comparability:** Some adjustment for demographics and clinical factors. ** Outcome:** CSF biomarkers and MP quantification performed using validated laboratory methods.
Xu et al. (2025)	Case control	3.5	1	3	7.5	**Selection:** Small PD *vs* control cohort; exposure measured using validated MP assays. ** Comparability:** Limited confounder control. ** Outcome:** Objective UPDRS scoring; integrated *in vitro* mechanistic validation.

B. Quality assessment of the included studies using ToxRTool for *in vitro* studies.									
Study (short)	**Main model**	**TS (0–5)**	**CS (0–6)**	**SD (0–4)**	**RD (0–2)**	**PL (0–1)**	**Total /18**	**ToxRTool category**	**Summary justification**
Huang et al. (2023) (PS/PE-NPs & dopaminergic SH-SY5Y)	Differentiated SH-SY5Y (dopaminergic)	4	5	4	2	1	16	Reliable ** without restrictions**	Commercial PS-/PE-NPs with size and vendor reported; cell line, differentiation protocol, medium and controls clearly described; realistic dose range based on human MNP levels; *n* = 3 per group; CCK-8, PI, ROS, mitochondrial, and rescue experiments with proper stats. Passage number not reported.
Wang et al. (2017) (BPA & SH-SY5Y/PC-12)	SH-SY5Y & PC-12	4	4	4	2	1	15	Reliable ** without restrictions**	BPA named with purity >99% and vendor; DMEM + 10% FBS, ATCC cell lines specified; exposure range 0–2,000 nM; multiple endpoints (Ca^2+^, ROS, ΔΨm, ATP, WB) with appropriate statistics. CAS number and passage not stated, and explicit positive controls not highlighted.
Tang et al. (2022) (PS-NPs & autophagy in SH-SY5Y)	SH-SY5Y	4	5	4	2	1	16	Reliable ** without restrictions**	50 nm PS-NPs from named supplier with size & form; SH-SY5Y origin (ATCC), culture conditions and PS-NP working concentrations (20–500 mg/L) fully described; negative controls + NAC and tBHP as functional controls; multiple cytotoxicity/ROS/mitochondrial/autophagy endpoints with clear methods and stats. Passage not given.
Jeong et al. (2024) (PS-NPs in C. elegans + A53T SH-SY5Y)	A53T α-syn-EGFP SH-SY5Y	4	5	4	2	1	16	Reliable ** without restrictions**	PS-NP beads, concentration (15 μ g/L), exposure duration and renewal scheme clearly reported; human A53T SH-SY5Y line source, DMEM + 10% FBS and incubation conditions described; multiple behavioral and α-syn aggregation endpoints with replicated experiments and defined analysis parameters. Passage and NP purity not explicitly stated.
Di Credico et al. (2023) (bisphenols & PFAS in human mDANs)	Stem cell–derived human midbrain DA neurons	4	5	4	2	1	16	Reliable ** without restrictions**	Test compounds (BPA/BPS/PFOS/PFOA, *etc.*) clearly defined and within “real-life” range (0.01–2 μ M); detailed differentiation protocol and treatment section; high-content imaging with 126 features, n and stats clearly defined; ML classification built on robust experimental data. Some details (passage/mycoplasma) not reported.
Gou et al. (2024) (PS-NPs, Aβ aggregation & neurotoxicity)	Aβ40/42 aggregation + neuronal cell assays	5	4	4	2	1	16	Reliable ** without restrictions**	PS nanoplastics (size, hydrophobic surface, vendor) and Aβ peptides well-characterized; in-vitro aggregation kinetics and neurotoxicity assays with appropriate controls; exposure concentrations justified as “low-dose”; clear dose–response and mechanistic plausibility. Cell system details are adequate but less extensive (*e.g.*, passage not given).
Cho et al. (2024) (New 8 Cho cell culture)	Human endothelial BBB model	5	5	4	2	1	17	Reliable without restrictions	Microparticles (0.2–1.0 μ m) fully characterized; human endothelial BBB model described; TEER, permeability, uptake & viability assays with replicates; biologically plausible mechanistic findings.
Ban, Shimoda & Chen (2021) (PS-NPs & SH-SY5Y)	Differentiated SH-SY5Y	5	6	4	2	1	18	Reliable ** without restrictions**	PS-NPs: size, shape, surface charge, wt% and synthesis route (surfactant-free) reported; SH-SY5Y source, medium, differentiation protocol, passage number (P15), seeding densities and variability explicitly documented; live/dead staining with positive “dead” controls, triplicate experiments and defined stats; strong biological plausibility and clear acknowledgment of limitations.
Monikh et al. (2024) (PS/PVC NPs & in-vitro BBB)	iPSC-derived endothelial cells + astrocytes (BBB model)	5	5	4	2	1	17	Reliable ** without restrictions**	PS/PVC nanoplastics doped with Gd as tracers; physicochemical characterization and biocorona composition (proteins/lipids) extensively reported; iPSC-derived BBB co-culture described; exposure (2 h, 5 μ g L^−1^) with quantification of uptake and transcytosis; robust analytics and dose interpretation; outcomes mechanistically consistent with nanoparticle transport/toxicokinetics. Passage not reported.

**Notes.**

NOS Score: Low risk of bias (8–9), Moderate risk (6–7) High risk (≤5).

NHANES National Health and Nutrition Examination Survey BPA Bisphenol A CSF Cerbrospinal fluid MPr Microparticles AD Alzheimer disease MCI Mild cognitive impairment MP microplastic Py-GC/MS Pyrolysis-Gas Chromatography-Mass Spectrometry TEM Tansmission Electron Microscopy PD Parkinson’s disease QA/QC Quality Assurance/Quality Control UPDRS Unified Parkinson’s Disease Rating Scale PS Plastics PE-NP Polystyrene-nanoplastics MNP Microplastics and nanoplastics CCK-8 Cell counting kit-8 PI Propidium iodide ROS Reactive Oxygen Species DMEM Dulbecco’s Modified Eagle Medium FBS Fetal Bouvine Serum ATCC American Type Culture Collection ATP Adenosine Triphosphate WB Western Blot PS-NP Polystyrene nanoplastic NAC N-acetylcysteine tBHP tert-Butyl hydroperoxide NP Nanoparticles PFAS Perfluoroalkyls mDANs mouse Dopaminergic Neurons DA Dopamine BPS Bisphenols PFOS Perfluorooctane sulfonate PFOA Perfluorooctanoic acid ML Machine Learning BBB Blood-brain barrier TEER Transepithelial Electrical Resistance wt% Weight by percent PVC Polyvinyl chloride Gd Gestational Day iPSC induced Pluripotent Stem Cell

## Results

### Search outcomes

From the selected 18 studies, eight were cross-sectional studies, one postmortem human organ analysis. In addition to nine *in vitro* cell culture studies. The final studies included dates between 2015 and 2025 and were published by authors from China, Brazil, Italy, South Korea, Germany, Canada, Japan, and the United States. The included studies and their characteristics are summarized in Table 2.

**Table 2:** Characteristics of included studies with limitations.

A. Clinical and epidemiological studies					
**Author, Year**	**Design**	**Population/Sample size**	**Exposure type**	**Outcome measure**	**Limitations**
Weng et al. (2022)	Cross-sectional (NHANES)	Older adults (*n* = 835)	Urinary phthalates	Cognitive function tests	Cross-sectional design; exploratory mixture models; no causal inference
Shi et al. (2023)	Cross-sectional (NHANES)	Older adults (*n* = 961)	Bisphenols & parabens	Delayed/instant recall	Single-spot urine; short-lived exposures; no causal inference
Magalhães et al. (2019)	Case–control	AD, MCI, controls (*n* = 43)	Plasma MPs	MMSE; FAST	Very small sample; convenience sampling; residual confounding
Landolfi et al. (2017)	Case–control	PD *vs* controls (*n* = 128)	Free *vs* conjugated BPA	BPA metabolism markers	Observational; modest sample; unmeasured confounders
Xu et al. (2025)	Case–control + in vitro	PD *vs* controls (*n* = 33)	Blood MPs (PVC, PP, PA66)	UPDRS; α-synuclein	Single-center; one-time exposure; limits extrapolation
Zheng et al. (2025)	Cross-sectional	Children 7–10 yrs (*n* = 5,670)	Urinary MPs	Working memory; attention	Short-term exposure; limited home/environment factors
He et al. (2025)	Memory-clinic cohorts	Amyloid+/–adults	CSF MPs	Aβ42, tau, MMSE	Modest sample sizes; cross-sectional; residual confounding
Xie et al. (2024)	Cross-sectional	Adults with varied BBB integrity (*n* = 28)	CSF MNPs	Albumin index; IL-6/IL-8	Small sample; infection effects confounded
Nihart et al. (2025)	Postmortem analysis	Human decedents	Tissue MPs	Brain MP burden	Single sample per organ; storage variability; associative only

B. In vitro and mechanistic studies				
**Author, Year**	**Model**	**Exposure type**	**Outcome measure**	**Limitations**
Huang et al. (2023)	SH-SY5Y neurons	Polystyrene nanoplastics	Mitochondrial dysfunction; mitophagy	Single polymer; immortalized neurons; high doses
Wang et al. (2017)	Neuronal cells	Bisphenol A	Insulin signaling; tau phosphorylation	Acute exposure; lacks systemic model; high BPA levels
Tang et al. (2022)	SH-SY5Y neurons	Polystyrene nanoplastics	Autophagy disruption; oxidative stress	Single cell line; short duration; no in vivo support
Jeong et al. (2024)	SH-SY5Y neurons	Nanoplastics	α-synuclein aggregation	In vitro only; limited polymer relevance
Di Credico et al. (2023)	Human dopaminergic neurons	BPA, BPS, PFAS	Neurite alterations; α-synuclein	Selected leachates; unclear dose relevance
Gou et al. (2024)	Neuronal cultures	Polystyrene nanoparticles	Aβ aggregation; neurotoxicity	High-dose; no BBB model; low environmental relevance
Ban, Shimoda & Chen (2021)	SH-SY5Y & human neurons	Micro/nanoplastics	ROS; viability changes	Limited polymers; in vitro only
Monikh et al. (2024)	Microfluidic BBB model	Nanoplastics	BBB translocation/biotransformation	Simplified BBB; lacks immune components
Cho et al. (2024)	3D BBB model	PS microplastics	BBB permeability; cytotoxicity	Only polystyrene; high-dose; lacks full neurovascular unit

**Notes.**

NHANES National Health and Nutrition Examination Survey AD Alzheimer’s disease MCI Mild cognitive impairment MPs Microparticles MMSE Mini-Mental State Examination FAST Functional Assessment Staging PD Parkinson’s disease BPA Bisphenol A PVC Polyvinyl chloride PP Polypropylene PA66 Polyamide 66 UPDRS Unified Parkinson’s Disease Rating Scale CSF Cerbrospinal fluid BBB Blood-brain barrier MNPs Microplastics and nanoplastics BPS Bisphenols PFAS Perfluoroalkyls ROS Reactive oxygen species

### Overview of clinical studies

In this review we look at nine human studies which looked at the association between microplastics (MPs), phthalates, bisphenol, and neurological outcomes. We looked at five cross sectional epidemiological studies, two case-control studies, a randomised clinical trial, and also two clinical mechanistic studies which looked at cerebrospinal fluid and postmortem brain tissue.

### Synthesis of findings

Because the included studies examined different exposure markers (bisphenol A, phthalates, microplastics/nanoplastics) and heterogeneous outcome domains (cognitive performance, neurodegenerative biomarkers, oxidative stress, and cellular injury), results were synthesized using a structured, subgroup-based approach.

### Human observational studies

#### Cognitive performance outcomes

Studies assessing bisphenols, phthalates, or urinary microplastics in relation to memory, attention, and executive function were synthesized together as a distinct subgroup. Across these studies, higher internal exposure to endocrine-disrupting chemicals or microplastics was consistently associated with poorer cognitive performance, particularly impaired delayed recall and working memory (Shi et al., 2023; Magalhães et al., 2019; Zheng et al., 2025; He et al., 2025; Landolfi et al., 2017). Because these studies were comparable in design and outcome type, they were summarized as one analytical subgroup; however, no effect sizes were pooled.

### Neurodegenerative biomarkers

A separate subgroup included studies linking MPs, bisphenols, or polymer fragments with Alzheimer’s disease–related biomarkers (Aβ42, tau), Parkinson’s disease markers, and functional neurodiagnostic measures. These studies were synthesized independently from cognitive-performance research due to differences in outcome measurement and underlying biological pathways. Within this subgroup, higher levels of microplastics or bisphenol exposure were associated with altered amyloid/tau profiles, increased α-synuclein, and worse functional scores (Unified Parkinson’s Disease Rating Scale; UPDRS) (Xu et al., 2025).

### *In-vitro* mechanistic studies

These experiments demonstrated pathways involving mitochondrial dysfunction, oxidative stress, autophagy disruption, Aβ aggregation, and α-synuclein misfolding, supporting mechanistic links but not providing epidemiologic effect estimation.

### Microplastics and phthalate exposure and cognitive function in community populations

Two large scale cross sectional NHANES based studies which reported that there is a very present association between exposure to plastic related toxins in terms of urine or blood and also reduced cognitive performance. Weng et al. (2022) reported that mixed phthalate exposure had a negative association with immediate and delayed recall scores and that MnBP and MBzP had the greatest role in that mix. Also Shi et al. (2023) reported similar negative associations for bisphenol A and parabens which in particular affected delayed memory performance in older adults and also they saw that in males the effect was greater.

### Microplastics burden in neurodegenerative diseases

Another two case control studies which looked at blood microplastics and bisphenol levels that there were in fact very large differences between neurodegenerative disease patients and healthy controls.

Landolfi et al. (2017) found that Parkinson’s disease patients exhibited higher blood bisphenol A (free fraction) and impaired bisphenol A (BPA) metabolism compared with healthy controls (Xu et al., 2025). Magalhães et al. (2019) demonstrated that platelet, leukocyte, endothelial, and neuron-derived microparticles were significantly elevated in Alzheimer’s disease (AD) and mild cognitive impairment, correlating with lower Mini-Mental State Examination (MMSE) z-scores and poorer functional status (Functional Assessment Staging (FAST) stages).

A more recent study by Xu et al. (2025) revealed substantially higher blood microplastics concentrations in Parkinson’s disease (PD) patients (notably polyvinyl chloride (PVC), polypropylene (PP), polyamide 66 (PA66)), with *in vitro* assays showing induction of dopaminergic apoptosis and p-α-synuclein accumulation, suggesting a mechanistic link to PD-related neurodegeneration.

### Evidence of microplastics penetration into the human central nervous system

Two clinical mechanistic studies provided direct evidence of MP accumulation in the central nervous system (CNS). Xie et al. (2024) detected polystyrene, polyethylene, polypropylene, and PVC particles in human cerbrospinal fluid (CSF), with significantly higher levels observed in individuals with blood–brain barrier impairment. Accumulated MPs did not significantly correlate with CSF IL-6 or IL-8 levels, suggesting non-inflammatory accumulation patterns. A landmark postmortem investigation by Nihart et al. (2025) demonstrated the presence of micro- and nanoplastics in human frontal cortex tissue using pyrolysis–gas chromatography-mass spectrometry (pyrolysis-GC/MS) and electron microscopy, with higher polyethylene burdens in brains compared to other organs, and markedly greater accumulation observed in decedent brains with diagnosed dementia.

Postmortem studies evaluating microplastics accumulation in brain tissue were synthesized separately, as they represent a different evidence domain with exposure quantification at the organ level rather than systemic biomarkers or urine measurements. These studies consistently showed higher microplastics burden in dementia brains and accumulation patterns favoring neural tissues over liver/kidney (Nihart et al., 2025).

### *In vitro* cell line studies

A total of nine *in-vitro* studies investigated the direct cellular effects of microplastics and nanoplastics (MNPs) using human neuronal, dopaminergic, or blood-brain barrier (BBB)-associated cell lines. Despite differences in particle size, polymer type, and exposure concentrations, these studies collectively demonstrate a consistent mechanistic pattern involving (1) mitochondrial damage, (2) dysregulated autophagy/mitophagy, (3) oxidative stress–driven apoptosis, and (4) enhanced neurodegenerative protein aggregation.

### Neuronal cells mitochondrial damages

Across differentiated SH-SY5Y neuroblastoma cells, exposure to polystyrene nanoplastics (PS-NPs) produced a rapid decline in metabolic activity, rising cytotoxicity, and significant reductions in mitochondrial membrane potential. Huang et al. (2023) showed that 50-nm PS-NPs directly accumulated inside mitochondria, causing severe morphological disruption, adenosine triphosphate (ATP) depletion, and impaired complex-I–mediated respiration. These functional defects were not rescued by antioxidant treatment, indicating a mechanism partly independent of classical reactive oxygen species (ROS) pathways. Wang et al. (2017) demonstrated parallel mitochondrial vulnerability in SH-SY5Y cells exposed to bisphenol A (a microplastics-associated monomer), reporting strong inhibition of IR/IRS-1/AKT/GSK-3β signaling and increased Alzheimer’s-related tau and APP fragments, linking plastic-derived chemicals to insulin-resistance–mediated neurotoxicity.

Tang et al. (2022) confirmed profound mitochondrial dysfunction, showing elevated Ca^2^^+^ influx, mitochondrial depolarization, and decreased ATP production following PS-NP exposure. Ban, Shimoda & Chen (2021) similarly observed mitochondrial swelling, disrupted neurite extension, and nuclear fragmentation at higher concentrations, with toxicity exceeding that of acrylamide, a known neurotoxin.

### Autophagy and mitophagy dysregulation

Multiple studies converged on autophagy/mitophagy activation as a central pathway. Huang et al. (2023) demonstrated that PS-NP–induced mitochondrial injury triggered excessive mitophagy *via* AMPK/ULK1 activation, worsening neuronal energy collapse and promoting cell death. Tang et al. (2022) showed significant upregulation of autophagy markers (LC3-II, Beclin-1, Atg5/12/16L), with RNA-silencing of Beclin-1 partially rescuing viability, confirming autophagy as a causal mediator rather than a compensatory response. This sustained mitophagic flux likely reflects an overburdened mitochondrial quality-control process, pushing neurons toward apoptosis.

### Oxidative stress, apoptosis, and cellular stress pathways

Oxidative stress emerged as a major upstream driver of cytotoxicity. Tang et al. (2022) observed sharp increases in ROS, cytochrome-c release, and caspase-3/9 activation following PS-NP exposure, indicating mitochondrial-apoptotic pathway engagement. Ban, Shimoda & Chen (2021) corroborated these findings with cellular leakage, vacuolation, and oxidative injury in plastics (PS)-exposed neuroblastoma cells. These converging data show that MNPs induce a combined oxidative–mitochondrial crisis, resulting in impaired survival signaling, mitochondrial fragmentation, and programmed cell death.

### Promotion of neurodegenerative protein aggregation

Several studies provided mechanistic evidence linking microplastics to classical neurodegenerative pathways:

### α-Synuclein aggregation (Parkinson’s disease mechanisms)

Jeong et al. (2024) showed that nanoplastics increased α-synuclein aggregate burden by ∼50% in SH-SY5Y cells, worsening dopaminergic dysfunction, mirroring Parkinson’s-like phenotypes observed *in vivo*. Di Credico et al. (2023) reported that bisphenols and perfluoroalkyls leached from plastics significantly increased α-synuclein and tyrosine hydroxylase (TH) staining intensity, while simultaneously reducing MAP2-positive neurite length mimicking early PD neurodegeneration signatures in human dopaminergic neurons.

### Aβ aggregation (Alzheimer’s disease mechanisms)

Gou et al. (2024) demonstrated that even very low concentrations (100 pM) of polystyrene nanoplastics significantly accelerated the nucleation and oligomerization of Aβ40 and Aβ42, producing amplified neurotoxicity and membrane injury driven by hydrophobic surface interactions between PS and Aβ fragments. Wang et al. (2017) further supported Alzheimer’s-like processes by showing upregulation of amyloid precursor protein (APP) fragments, BACE-1, and phosphorylated tau species, directly linking bisphenol A exposure to Aβ and tau pathology.

### Blood–brain barrier penetration and translocation in *in-vitro* models

Monikh et al. (2024) demonstrated that both polystyrene and PVC nanoplastics “with or without a biological corona” were able to cross an induced Pluripotent Stem Cell (iPSC)-derived human BBB model, with PVC showing the higher transcytosis rate. Corona formation reduced cellular uptake but did not prevent barrier passage, suggesting that nanoplastics remain bioavailable to CNS tissues even after plasma conditioning.

## Discussion

The objective of this systematic review was to examine the potential correlation between neurodegenerative disease development and patient exposure to microplastics originating from dental sources. This is, as far as we are aware, the initial review to examine this phenomenon through the exclusive exclusion of animal studies and a focus on human studies. Chesnut et al. (2021) revealed that the dissimilarities between human and animal oligodendrocytes render animals unsuitable for modeling these disorders. Consequently, this review specifically omitted studies conducted on animals. Overall, despite variations in study design and measured outcomes, the articles consistently demonstrate that exposure to microplastics contributes to the onset of cognitive decline and neurodegenerative diseases, such as Alzheimer’s disease and Parkinson’s disease, or the pathological changes associated with these conditions.

Magalhães et al. (2019) found that there were higher levels of microplastics in patients with Alzheimer’s disease compared to healthy individuals. Microplastics were found to be associated with cognitive impairment and lower functional performance, and so exposure is involved in the pathophysiology of degenerative dementia. Shi et al. (2023) and Weng et al. (2022) both found similar findings in their studies, in which they found that the exposure to microplastics, namely bisphenol A results in lower cognitive function. In addition, Maserejian et al. (2012) found poorer neurophysiological test scores in children with greater exposure to resin-based composite that leaches the microplastics bisphenol A.

Landolfi et al. (2017) proposed a potential mechanism for this association, based on their case-control study. They found that bisphenol A might contribute to dopaminergic toxicity and the development of Parkinson’s disease. This was supported by their observation that patients with Parkinson’s disease had reduced glucuronidation of bisphenol A, indicating impaired metabolism compared to the control group. Although a significant portion of bisphenol A (BPA) in humans is quickly metabolized, leading to a reduction in the generation of harmful metabolites, the remaining BPA that is not metabolized enhances the synthesis of reactive oxygen species. This stimulation occurs through the enzymatic and nonenzymatic formation of phenoxyl radicals (Engin & Engin, 2021).

**Figure 2:**
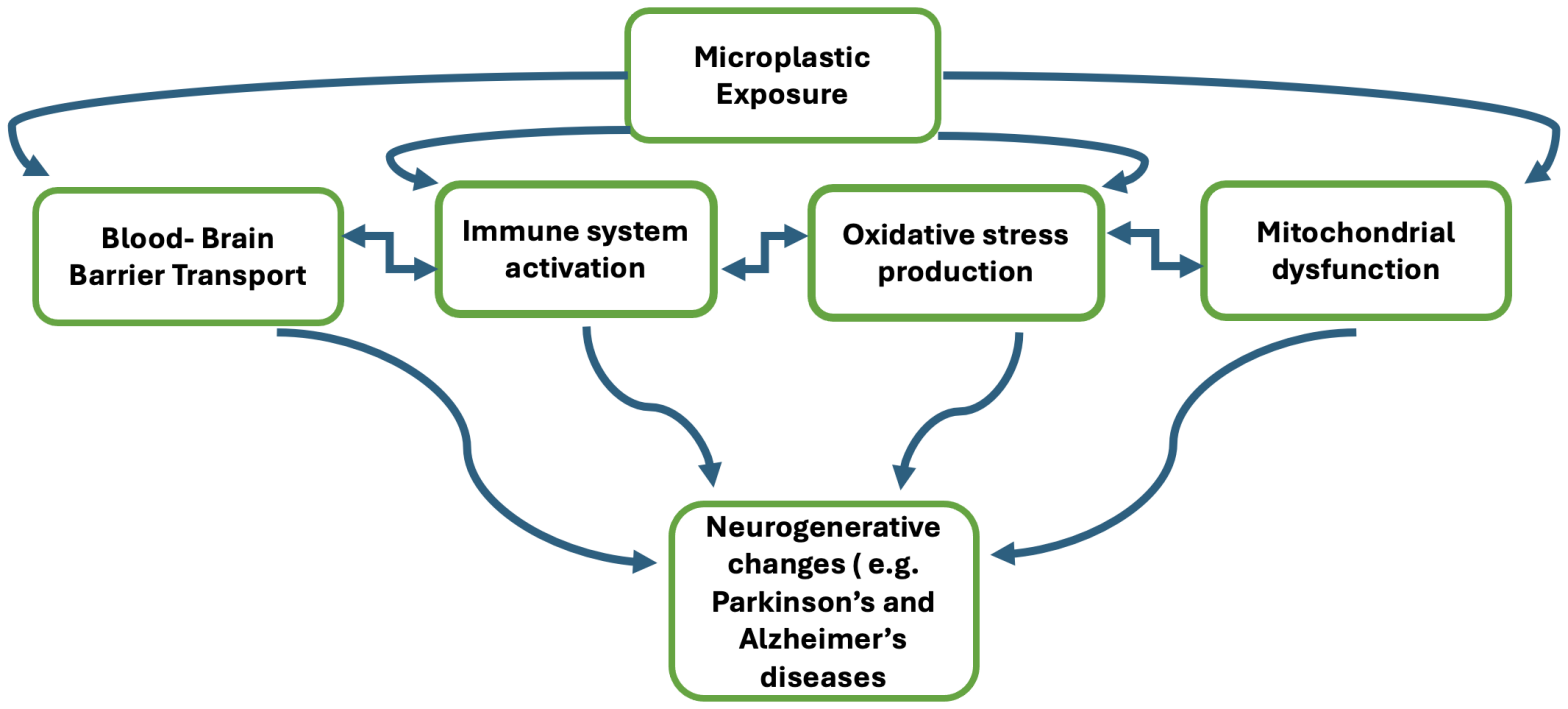
Proposed biological pathways of microplastic exposure and neurodegeneration.

Shapiro & Katchur (2022) suggested that the potential human body reaction to exposure to microplastics can occur through four pathways: (a) transportation to the brain, leading to the activation of neurotoxicity, (b) entry into the immune system, triggering an inflammatory response upon detection by immune cells, (c) induction of oxidative stress, and (d) disruption of mitochondrial function. Neurodegenerative disorders involve the development and pathophysiology of conditions where neurotoxicity, inflammatory response, and oxidative stress cause damage to dopaminergic neurons. This damage leads to neuronal cell death and a decrease in the overall number of dopaminergic neurons. Figure 2 summarized the proposed pathways.

After ingestion, microplastics can reach the gut, travel through the lymph and circulatory system, eventually reaching to the brain and crossing the blood–brain barrier (Protyusha, Kavitha & Robin, 2024; Ding et al., 2018). Neurotoxicity results from the detrimental effects of microplastics such as bisphenol A on the blood–brain barrier integrity (Engin & Engin, 2021). Neurons are susceptible to microplastic exposure once they have traversed the blood–brain barrier (Daneman & Prat, 2015). The presence of microplastics could potentially harm neurons and neurological processes if they do induce inflammation in the brain (Ding et al., 2018). Enhancing the inflammatory response, microplastics may stimulate the secretion of proinflammatory cytokines (Varon & Shai, 2015). Another fundamental characteristic of neurodegenerative diseases and dementia, including Alzheimer’s disease, has been proposed to be inflammation. A diverse array of proinflammatory cytokines, reactive oxygen species, and nitric oxide are secreted by chronically activated microglia. Numerous investigations of postmortem tissues from Alzheimer’s disease patients have identified the inflammation as a link between amyloid-β plaques and neurofibrillary tangles (Magalhães et al., 2019; Chakraborty et al., 2023; McCarthy et al., 2017; Ramirez-Bermudez, 2012). Neuroinflammation is not the cause, but rather an outcome of one or more risk factors linked to neurodegeneration. In addition, it contributes to the worsening of the illness by intensifying the β-amyloid and tau pathologies (Kinney et al., 2018).

The majority of research primarily concentrate on the neurotoxicity of microplastics alone, disregarding their combined actions with other biomolecules and the resulting neurotoxicity. According to Wang et al. (2017)’s research, (exposure to bisphenol A leads to insulin resistance in the brain, which might potentially contribute to the onset of neurodegenerative disorders. Brain insulin plays a vital role in maintaining the neuronal energy balance, regulating the cell proliferation of neurons, differentiation, the release of neurotransmitters, axonal growth, and preventing oxidative stress (Zhao & Townsend, 2009; Kwai et al., 2015). Insulin resistance has been shown to be a risk factor for metabolic disorders (Hoscheidt et al., 2016; Marmugi et al., 2014). The study showed notable disruptions in insulin levels and an elevation in the levels of various proteins associated with Alzheimer’s disease, including Aβ 1–42, α-CTF, β-CTF, BACE-1, amyloid precursor protein, and phosphorylated tau proteins (S404, S214, T205, S396, S199). These proteins are all involved in the development of Alzheimer’s disease. Bisphenol A significantly increases the expression of amyloid precursor protein. Physiologically, elevated amyloid precursor protein expression results in the buildup of amyloid-β peptides, which can form aggregates (Wang et al., 2017).

Bisphenol A hinders the activity of the insulin receptor pY1355, which is a phosphorylation site for a tyrosine kinase. This site plays a crucial role in initiating the signaling pathways for insulin (Coloma et al., 2000). The phosphorylation of serine residues on the insulin receptor S-1, which is a characteristic marker of insulin resistance, was significantly elevated (Wang et al., 2017; Jaffiol et al., 1999). In addition, bisphenol A significantly hinders the process of phosphorylation of protein kinase B (AKT) at serine 473, a crucial upstream signaling component that controls the deactivation of GSK-3β. It also decreases the level of phosphorylated GSK-3β at the serine nine residue, which indicates an increase in GSK-3β activity. Furthermore, it reduces the level of phosphorylated GSK-3α at the serine 21 residue, which indicates an increase in GSK-3α activity. The activities of GSK-3α and β, which contribute to the production of amyloid precursor protein, and the excessive phosphorylation of tau, have been seen to rise (Jaffiol et al., 1999). Consequently, exposure to bisphenol A leads to an increase in the expression of amyloid precursor protein and p-tau. Elevated amyloid precursor protein expression would result in a greater amount of material available for cleavage, leading to an increased likelihood of creating amyloid-β, a harmful polypeptide produced by the breakdown of amyloid precursor protein by BACE-1 (Chakraborty et al., 2023). BPA greatly enhanced the expression of BACE-1, leading to a potential elevation in the production of extracellular Aβ142 (Selkoe, 2001). The results also imply that exposure to BPA leads to an increase in the hydrolysis of amyloid precursor protein (APP), namely in the C-terminal fragments (CTF), such as β-CTF and α-CTF. These findings suggest that disruptions in the “IR/IRS-1/AKT/GSK-3α/APP” axis play a crucial role in the neurotoxic effects of bisphenol A, which resemble those seen in Alzheimer’s disease (Wang et al., 2017). Moreover, amyloid plaques consist of fibrillar aggregates generated by the amyloid-β peptide and are located outside of cells. The senile plaques detected in the brains of individuals with neurodegenerative diseases consist primarily of these plaques (Martin-Rapun et al., 2017). The amyloid-β peptide consists of 35 to 42 amino acids and is primarily observed in the conformation of a random coil (Murphy & LeVine III, 2010). Gou et al. (2024) discovered that amyloid-β peptide, by forming aggregates, is the primary pathogenic protein responsible for neurotoxicity in Alzheimer’s disease. This peptide is produced from a specific type of protein called APP that is located in the cell membrane. The production of this peptide occurs through a pathway known as the amyloidogenic pathway. In this pathway, amyloid precursor protein is enzymatically processed by two membrane-bound endoproteases, called β-secretase and γ-secretase. This processing results in the formation of amyloid-β peptide, which can have different structures at its C-terminal end (Chen et al., 2017; O’Brien & Wong, 2011). The peptides ending at position 40 (amyloid-β1-40) and position 42 (amyloid-β1-42) are the most prevalent among the several amyloid-β types. Amyloid-β1-42 is more prone to forming fibrils and has a higher hydrophobicity than Aβ1-40. It is the main kind of amyloid deposited in the brain (Selkoe, 2001). At present, the main cause of neurodegenerative disorders such as Alzheimer’s disease is believed to be the formation of toxic, prefibrillar oligomers from amyloid-β peptides (Walsh & Selkoe, 2004).

Wang et al. (2017) proposes that the sequential process involves the disruption of insulin signaling pathways due to exposure to microplastics. This disruption is characterized by a “decrease in insulin receptor tyrosine phosphorylation and an increase in IRS1 serine phosphorylation, ultimately resulting in lower AKT phosphorylation. AKT inactivation leads to the excessive activation of GSK3α and GSK3β, which are crucial enzymes involved in the synthesis of amyloid precursor protein and p-tau. Amyloid precursor protein is then broken down by BACE-1, which promotes the production of Aβ1–42, and the higher levels of Aβ1–42 and the amplification of p-tau lead to the development of a condition similar to Alzheimer’s” (Huang & Feng, 2025). In their research, Wang et al. (2017), Tang et al. (2022) and Huang & Feng (2025) also discovered that microplastics induce mitochondrial injury by significantly increasing lactate dehydrogenase release, inhibiting cell proliferation, and producing an excessive amount of reactive oxygen species. The increase in levels can stimulate the oxidative stress response, which is closely linked to the onset of neurodegeneration and could be involved in the pathological pathways of neurodegenerative disease (Huang et al., 2023; Wang et al., 2017; Tang et al., 2022). Mitochondrial dysfunction is evidenced by the suppression of cellular growth, heightened release of lactate dehydrogenase, activation of oxidative stress reactions, high levels of calcium ions, programmed cell death, and diminished mitochondrial membrane potential and adenosine triphosphate level (Tang et al., 2022). Costa & Cairrao (2024) also confirmed this in their research. They found that six hours of exposure to bisphenols could increase reactive oxygen species levels, while 24 and 48 h of exposure could induce increased rates of apoptosis and lactate dehydrogenase leakage. Additionally, seven days of exposure could inhibit cell proliferation. Reactive oxygen species accumulation is partially responsible for the initiation of cytotoxicity and promotes oxidative stress-induced apoptotic signaling and eventual apoptosis, indicating the potential role of microplastics in the development of neurodegenerative diseases and Alzheimer’s-like pathological changes (Costa & Cairrao, 2024). Oxidative stress is defined as an interrupted equilibrium between the body’s ability to detoxify reactive oxygen species and production of reactive oxygen species, causing cellular damage (Dias, Junn & Mouradian, 2013). Mitochondria generate reactive oxygen species by oxidative phosphorylation, a process that links adenosine triphosphate production with electron leakage (Bottje, 2019). Within the central nervous system, glial cells’ mitochondria generate an excessive amount of reactive oxygen species, leading to oxidative stress and subsequent protein damage. These impaired proteins can subsequently regulate pathways outside of cells and activate processes that promote inflammation. The brain’s inflammatory response can be triggered by these specific pathways (Taylor, Main & Crack, 2013). In Parkinson’s disease, dopamine undergoes oxidation readily, leading to the formation of reactive oxygen species. Thus, the presence of reactive oxygen species might trigger death in dopaminergic neurons, leading to a reduction in their quantity (Weng et al., 2022). Following the activation of apoptotic signaling caused by oxidative stress, apoptotic cells ultimately experience secondary necrosis and discharge their contents, including lactate dehydrogenase, into the surroundings. Lactate dehydrogenase is an intracellular enzyme that is confined to the cytoplasm and is unable to traverse the cell membrane. When a cell is destroyed or undergoes necrosis, it is released into the extracellular matrix. The rate at which this release occurs reflects the integrity of the cell membrane and is commonly used as an indication of the extent of cell damage (Tang et al., 2025).

Neurotoxicities are observed in the course of neurodegenerative disorders, such as Parkinson’s disease, where the reduction of dopamine is associated with the disease and is responsible for the characteristic motor dysfunction observed in patients (Tolosa et al., 2021). The alpha-synuclein aggregates caused by microplastic exposure can disrupt normal cellular processes and inhibit the activity of tyrosine hydroxylase, the enzyme that controls the rate of dopamine generation in the brain. Both Jeong et al. (2024) and Di Credico et al. (2023) corroborated this finding in their respective research. The accumulation of alpha-synuclein aggregates is responsible for the development of distinct motor symptoms, including bradykinesia, stiffness, tremors, as well as non-motor symptoms including cognitive impairment, mental abnormalities, and sleep difficulties. These results have resemblance to Parkinson’s disease since individuals with Parkinson’s exhibit reduced amounts of dopaminergic and glutamatergic neurons, as well as motor dysfunction. These findings indicate that the presence of microplastics leads to the development of neurotoxic effects, perhaps playing a role in the observed disease progression in individuals with Parkinson’s disease (Shapiro & Katchur, 2022). By comprehending the mechanisms *via* which microplastics impact the body and contribute to neurodegenerative illnesses, the peril posed by microplastics may be acknowledged and promote the adoption of preventive measures to reduce patients’ exposure to microplastics.

The limitation of this systematic review is mainly the small number of articles included in the study. There is a limited amount of clinical research and studies conducted on the human population, with a focus on animal studies, and so many of the articles found from the databases were excluded. Nonetheless, the included studies in this review show invaluable research and merit, and the results and conclusions should be taken into consideration. A quantitative meta-analysis was not feasible because the included studies differed substantially in methodology and reporting, and many did not provide extractable effect estimates. Pooling these heterogeneous data would therefore have produced misleading results.

In addition, while the primary focus of the systematic review was to examine the impact of dental microplastics, it is important to note that the findings may exhibit some variation since various types of microplastics can have distinct impacts on the human body.

Publication and language bias should also be considered when interpreting these findings. Because the review included only English-language publications, relevant studies published in other languages may have been missed, potentially limiting the global scope of the evidence. In addition, the reliance on peer-reviewed literature raises the possibility of publication bias, as studies reporting null or negative associations may be less likely to be published. These factors may influence the completeness and balance of the available evidence.

While associations between various polymers and neurological outcomes have been reported in environmental and toxicological studies, the presence of these polymers within dental materials and their potential exposure pathways in the human body require further investigation through dedicated, dental-specific research. Well-designed studies evaluating microplastic release from dental materials are needed to clarify real-world exposure levels. In addition, future meta-analyses will be valuable for strengthening these observations once a sufficient number of methodologically comparable studies become available.

## Conclusion

This review indicates a possible association between microplastics-related exposures and changes in cognitive outcomes, neurodegenerative biomarkers, and cellular mechanisms relevant to neurological health. However, the evidence is largely based on cross-sectional and *in-vitro* studies, preventing any causal interpretation. Standardized, longitudinal human research is needed to clarify exposure–response relationships and determine the clinical significance of these findings. Given the widespread use of polymer-based materials, including those in dental applications, continued monitoring of material safety and potential long-term systemic effects remains important. Future work integrating epidemiologic and mechanistic data will be essential to guide safer material development and regulatory decisions.

Given the widespread use of resin-based dental materials, there is a pressing need to develop standardized methods for quantifying their microplastics and nanoplastic release, as well as validated biomonitoring approaches in human populations, to better evaluate exposure levels and associated systemic risks.

##  Supplemental Information

10.7717/peerj.20829/supp-1 Supplemental Information 1 Prisma Checklist

10.7717/peerj.20829/supp-2 Supplemental Information 2 Raw data

10.7717/peerj.20829/supp-3 Supplemental Information 3 Database search strings

10.7717/peerj.20829/supp-4 Supplemental Information 4 Full 18-Item ToxRTool Scoring Matrix for in vitro studies

## Additional Information and Declarations

### Data Availability

The following information was supplied regarding data availability: This is a systemic review/meta-analysis.
