# Supplementary material for: Dental microplastics as emerging neurotoxicants: a systematic review on human data
Source: PeerJ. 2026 Feb 26;14:e20829. doi: 10.7717/peerj.20829 (PMC12950184; doi:10.7717/peerj.20829)
Supplement: Supplemental Information 3 [file peerj-14-20829-s003.docx]

Database search strings

| Database / Platform | Search String | Limits / Filters |
| --- | --- | --- |
| PubMed (NCBI) | [Microplastic [mesh terms] OR (microplastics) OR (nanoplastic)) AND (dental materials [mesh terms] (material, dental) OR (composite) OR (bisphenol A) OR (bis-GMA) OR (BPA) OR (phthalate)) AND (Neurodegenerative disease [mesh terms] OR (Parkinson’s) OR (Alzheimer’s disease) OR (neurotoxicity) OR (cognitive degeneration) | Humans filter; date & English language filter |
| MEDLINE (Ovid) | *(microplastic*.tw,kf. OR micro-plastic*.tw,kf. OR microbead*.tw,kf. OR exp Microplastics/ OR exp Nanoparticles/ OR nanoplastic*.tw,kf. OR "plastic particle*".tw,kf. OR exp Phthalic Acids/ OR phthalate*.tw,kf. OR exp Bisphenol A/ OR bisphenol A.tw,kf. OR BPA.tw,kf. OR bis-GMA.tw,kf.) AND (exp Dental Materials/ OR exp Composite Resins/ OR dental material*.tw,kf. OR resin-based composite*.tw,kf.) AND (exp Neurodegenerative Diseases/ OR exp Alzheimer Disease/ OR exp Parkinson Disease/ OR neurodegenerat*.tw,kf. OR neurotox*.tw,kf. OR dementia.tw,kf.) NOT (exp Animals/ NOT Humans/)* | Ovid Humans filter applied |
| Scopus (Elsevier) | *(TITLE-ABS-KEY(microplastic* OR "micro-plastic*" OR microbead* OR nanoplastic* OR "plastic particle*" OR "plastic debris" OR phthalate* OR "bisphenol A" OR BPA OR bis-GMA) AND TITLE-ABS-KEY("dental material*" OR "resin-based composite*" OR composite* OR compomer* OR "oral healthcare products" OR toothpaste) AND TITLE-ABS-KEY(neurotox* OR neurodegenerat* OR dementia OR Alzheimer* OR Parkinson* OR "cognitive impairment"))* | Human filter |
| EBSCOhost — CINAHL Complete | *((microplastic* OR micro-plastic* OR microbead* OR nanoplastic* OR "plastic particle*" OR phthalate* OR "bisphenol A" OR BPA OR bis-GMA) AND ("dental materials" OR "resin-based composites" OR composite* OR toothpaste OR "oral health products") AND (neurotox* OR neurodegenerat* OR dementia OR Alzheimer* OR Parkinson* OR "cognitive impairment"))* | Peer-reviewed only , Humans filter |
| ScienceDirect (Elsevier) | *(microplastic* OR nanoplastic* OR "plastic particle*" OR "bisphenol A" OR BPA OR phthalate*) AND ("dental material" OR "resin composite" OR composite* OR toothpaste) AND (neurotoxicity OR "neurodegenerative disease" OR Alzheimer OR Parkinson OR "cognitive decline")* | Research articles; date limit |
