# Supplementary material for: Dental microplastics as emerging neurotoxicants: a systematic review on human data
Source: PeerJ. 2026 Feb 26;14:e20829. doi: 10.7717/peerj.20829 (PMC12950184; doi:10.7717/peerj.20829)
Supplement: Supplemental Information 4 [file peerj-14-20829-s004.docx]

**Supplementary table (1): Full 18-Item ToxRTool Scoring Matrix for in vitro studies**

| Study | TS1 | TS2 | TS3 | TS4 | TS5 | CS1 | CS2 | CS3 | CS4 | CS5 | CS6 | SD1 | SD2 | SD3 | SD4 | RD1 | RD2 | PL1 | Total |
| --- | --- | --- | --- | --- | --- | --- | --- | --- | --- | --- | --- | --- | --- | --- | --- | --- | --- | --- | --- |
| Wang 2017 | 1 | 0 | 1 | 1 | 1 | 1 | 1 | 1 | 0 | 1 | 0 | 1 | 1 | 1 | 1 | 1 | 1 | 1 | **15** |
| Huang 2023 | 1 | 1 | 1 | 0 | 1 | 1 | 1 | 1 | 0 | 1 | 1 | 1 | 1 | 1 | 1 | 1 | 1 | 1 | **16** |
| Tang 2022 | 1 | 1 | 1 | 0 | 1 | 1 | 1 | 1 | 0 | 1 | 1 | 1 | 1 | 1 | 1 | 1 | 1 | 1 | **16** |
| Jeong 2024 | 1 | 1 | 1 | 0 | 1 | 1 | 1 | 1 | 0 | 1 | 1 | 1 | 1 | 1 | 1 | 1 | 1 | 1 | **16** |
| Di Credico 2023 | 1 | 1 | 1 | 0 | 1 | 1 | 1 | 1 | 0 | 1 | 1 | 1 | 1 | 1 | 1 | 1 | 1 | 1 | **16** |
| Gou 2024 | 1 | 1 | 1 | 1 | 1 | 1 | 1 | 1 | 0 | 1 | 0 | 1 | 1 | 1 | 1 | 1 | 1 | 1 | **16** |
| Monikh 2024 | 1 | 1 | 1 | 1 | 1 | 1 | 1 | 1 | 0 | 1 | 1 | 1 | 1 | 1 | 1 | 1 | 1 | 1 | **17** |
| **Cho 2024** | 1 | 1 | 1 | 1 | 1 | 1 | 1 | 1 | 0 | 1 | 1 | 1 | 1 | 1 | 1 | 1 | 1 | 1 | **17** |
| Ban 2021 | 1 | 1 | 1 | 1 | 1 | 1 | 1 | 1 | 1 | 1 | 1 | 1 | 1 | 1 | 1 | 1 | 1 | 1 | **18** |

**Items (abbreviated)**

- **TS1**: Chemical/polymer name clearly stated
- **TS2**: Identifier (CAS / catalog / batch) reported
- **TS3**: Supplier/source reported
- **TS4**: Purity or composition reported
- **TS5**: Physical characterization (size/shape/surface or form/stability)
- **CS1**: Cell type & species given
- **CS2**: Cell origin/source (ATCC, lab etc.)
- **CS3**: Culture conditions (medium, serum, CO₂, etc.)
- **CS4**: Passage number / differentiation stage documented
- **CS5**: Negative/vehicle controls described
- **CS6**: Positive or reference controls (where relevant)
- **SD1**: Exposure concentrations described
- **SD2**: Exposure durations described
- **SD3**: Replicates/independent experiments stated
- **SD4**: Statistical methods described and appropriate
- **RD1**: Data with variability (e.g., mean±SD/SE, replicates)
- **RD2**: Clear dose–response / endpoint interpretation
- **PL1**: Biological plausibility & internal consistency

**1 = criterion clearly met**
**0 = not reported / not clearly met**
